# Complete Genome Sequence of an *mcr-9*-Possessing Enterobacter asburiae Strain Isolated from a Cat in Japan

**DOI:** 10.1128/MRA.00281-21

**Published:** 2021-07-01

**Authors:** Toyotaka Sato, Masaru Usui, Kazuki Harada, Yukari Fukushima, Chie Nakajima, Yasuhiko Suzuki, Shin-ichi Yokota

**Affiliations:** a Department of Microbiology, Sapporo Medical University School of Medicine, Sapporo, Japan; b Laboratory of Food Microbiology and Food Safety, Department of Health and Environmental Sciences, School of Veterinary Medicine, Rakuno Gakuen University, Ebetsu, Japan; c Department of Veterinary Internal Medicine, Tottori University, Tottori, Japan; d Division of Bioresources, Hokkaido University, Research Center for Zoonosis Control, Sapporo, Japan; e International Collaboration Unit, Research Center for Zoonosis Control, Hokkaido University, Sapporo, Japan; Loyola University Chicago

## Abstract

We report the complete genome sequence of the *mcr-9*-possessing strain Enterobacter asburiae En30, isolated from a cat in Japan. The genome sequence was obtained by using long- and short-read sequencing.

## ANNOUNCEMENT

Enterobacter asburiae is a bacterial species included in the Enterobacter cloacae complex (1) and isolated from the intestinal tracts and clinical specimens of humans and animals (2, 3). Some *E. asburiae* clinical isolates exhibit multidrug resistance (4), and the last-line drugs colistin and tigecycline are an option for treatment. *mcr-9*, like other *mcr* genes, encodes phosphoethanolamine transferase and reduces colistin susceptibility (5). *mcr-9*-harboring plasmids in the Enterobacter cloacae complex have been reported in human samples (6–8), while few have been reported in companion animals.

*E. asburiae* strain En30 was isolated by swabbing the nasal cavity of a cat in Japan in 2015, followed by isolation on CHROMagar Escherichia coli coliform (ECC) agar (Becton, Dickinson, NJ) and incubation overnight at 37°C. En30 was subcultured on the agar and kept at −80°C in 10% glycerol stock. Genomic DNA was extracted for short-read sequencing using the Wizard genomic DNA purification kit (Promega, Madison, WI). A DNA library was prepared using the Nextera XT kit and sequenced on the MiSeq platform (Illumina, San Diego, CA). The resulting 1,261,146 (300-bp, paired-end) reads were trimmed using fastp v0.20.1 (9).

Genomic DNA was extracted for long-read sequencing using the Genomic-tip 20/G and genomic DNA buffer set (Qiagen, Hilden, Germany). Library preparation was performed using the rapid barcoding sequencing kit SQK-RBK004 (Oxford Nanopore Technologies, Oxford, UK), following the manufacturer’s protocol. Sequencing was performed on the MinION with a FLO-MIN-106 R9.4 flow cell (Oxford Nanopore Technologies) using MinKNOW software for 48 h with no alterations to any voltage scripts. The obtained 440,000 reads (*N*_50_, 3,932 bp) were demultiplexed using Porechop v0.2.4 (https://github.com/rrwick/Porechop); then, the reads were adaptor trimmed and quality filtered using NanoFilt (Q score, 10; minimum length, 1,000 bp) (10).

The long reads were error corrected with the short reads using LoRDEC v0.6 (11). *De novo* assembly of the error-corrected long reads was performed using Flye v2.8 (12). The assembled contigs were error corrected with the short reads using Pilon v1.24 (13). Finally, the assembled sequences were annotated using DFAST v1.1.0 with standard settings (14). Software tools used default parameters unless otherwise indicated.

We obtained three different-sized complete genome sequences (Table 1). *mcr-9* was located on pEN30L (an IncHI2 plasmid) at nucleotide positions 160415 to 162034. pEN30L was 99.9% similar to the *mcr-9*-carrying plasmid, pIHI2-233, of Enterobacter hormaechei strain Y233 (GenBank accession number CP049047.1), derived from a patient in China using BLAST (8). pEN30L possessed other antimicrobial resistance genes as revealed by ResFinder v4.1 (Table 1) (15).

**TABLE 1:** Features of the En30 genome

Genome component	Name	Length (bp)	Coverage (×)	G+C content (%)	Antimicrobial resistance genes
Chromosome	En30 chromosome	4,722,066	82	55.9	*bla*_ACT-10_, *fosA*
Large plasmid	pEN30L	284,167	51	46.8	*mcr-9*, *aac(6')-Ib3*, *aph(6)-Id*, *bla*_TEM-1B_, *dfrA19*, *aac(6')-Ib-cr*, *catA2*, *tetD*
Small plasmid	pEN30S	85,172	161	52.7	*bla*_DHA-1_, *aac(3)-IIa*, *sul1*, *dfrA17*, *mph*(A), *qacE*, *qnrB4*

### Data availability.

The whole-genome sequence has been deposited at DDBJ/ENA/GenBank under the accession numbers AP024498 (chromosome), AP024499 (pEN30S), and AP024500 (pEN30L). The Illumina and MinION sequence reads were deposited in the Sequence Read Archive (SRA) database under the accession numbers DRR283416 and DRR283417, respectively.
